# Different Heterotrimeric G Protein Dynamics for Wide-Range Chemotaxis in Eukaryotic Cells

**DOI:** 10.3389/fcell.2021.724797

**Published:** 2021-08-03

**Authors:** Yoichiro Kamimura, Masahiro Ueda

**Affiliations:** ^1^Laboratory for Cell Signaling Dynamics, RIKEN, Center for Biosystems Dynamics Research (BDR), Suita, Japan; ^2^Laboratory of Single Molecule Biology, Graduate School of Frontier Biosciences, Osaka University, Suita, Japan

**Keywords:** chemotaxis, GPCR signaling, heterotrimeric G protein, gradient sensing, dynamic range

## Abstract

Chemotaxis describes directional motility along ambient chemical gradients and has important roles in human physiology and pathology. Typical chemotactic cells, such as neutrophils and *Dictyostelium* cells, can detect spatial differences in chemical gradients over a background concentration of a 10^5^ scale. Studies of *Dictyostelium* cells have elucidated the molecular mechanisms of gradient sensing involving G protein coupled receptor (GPCR) signaling. GPCR transduces spatial information through its cognate heterotrimeric G protein as a guanine nucleotide change factor (GEF). More recently, studies have revealed unconventional regulation of heterotrimeric G protein in the gradient sensing. In this review, we explain how multiple mechanisms of GPCR signaling ensure the broad range sensing of chemical gradients in *Dictyostelium* cells as a model for eukaryotic chemotaxis.

## Introduction

Chemotaxis describes the directional migration of cells in response to chemical gradients. It is required for many essential physiological processes including early embryogenesis, wound healing, immune responses (Sonnemann and Bement, 2011; Trepat et al., 2012; Nourshargh and Alon, 2014; de Oliveira et al., 2016; Shellard and Mayor, 2016; Norden and Lecaudey, 2019) and more. Conversely, aberrations of chemotaxis result in various pathological conditions, such as autoimmune diseases and cancer metastasis (Stuelten et al., 2018). *Dictyostelium discoideum* cells as well as their evolutionally distant cells, mammalian neutrophils, are common model systems for the study of chemotaxis mechanisms (Artemenko et al., 2014; Thomas et al., 2018). These cells move fast, a phenomenon known as amoeboid movement. The movement is propelled by the protrusion of plasma membrane filled with filamentous actin (f-actin), which leads to the structure known as the pseudopod. Cells spontaneously form pseudopods during random motility. However, chemical cues bias pseudopod formation to the front of the cells, which is defined as the membrane portion facing the higher concentration. The back of the cells ensures the directional motility by producing contractile forces from the action of myosin II on cortical f-actin. Therefore, cells have morphological anisotropy or polarity during chemotaxis. Eukaryotic chemotactic cells can decipher ambient chemical gradients by spatial sensing, whereas bacteria use temporal sensing (Parent and Devreotes, 1999; Tu and Rappel, 2018). Indeed, *D. discoideum* cells treated with latrunculin, an inhibitor of actin polymerization, only activate signaling molecules at their front side (Parent et al., 1998).

*D. discoideum* cells use chemotaxis in their lifecycle; for example, folate chemotaxis is used to forage for bacteria and chemotaxis to 3′,5′-cyclic adenosine monophosphate (cAMP), which *D. discoideum* cells secrete upon starvation to make multicellular structures that finally reach fruiting bodies (Artemenko et al., 2014). Mammalian neutrophils are recruited to a wide range of signals including N-formyl peptides, CXC-chemokine ligand 8 (CXCL8), leukotriene B_4_, and others (Nourshargh and Alon, 2014; de Oliveira et al., 2016). Furthermore, chemotaxis functions in very shallow gradients within broad ranges of background chemical concentrations. For example, *D. discoideum* cells can move in response to 2% cAMP gradients along their cellular length in a sub-μM to mM background concentration range (Mato et al., 1975; Fisher et al., 1989; Song et al., 2006; Ohtsuka et al., 2021). Similar quantitative properties have been observed in leukocyte chemotaxis (Zigmond, 1977). Chemical attractants associate with cognate receptors on the plasma membrane, triggering intracellular signal transduction, and the association simultaneously gives rise to receptor modifications (Hoeller et al., 2014; Tu and Rappel, 2018). Thereby, the receptors return to the pre-stimulated state in a process known as adaptation (Hoeller et al., 2014; Tu and Rappel, 2018). However, while true for bacteria, recent studies have shown chemotaxis mechanisms besides receptor adaptation are used by eukaryotic cells.

Studies on *D. discoideum* cells and neutrophils have formed a molecular framework for eukaryotic chemotaxis that is evolutionally conserved. G protein-coupled receptor (GPCR) signaling serves as a sensor of chemical gradients (Artemenko et al., 2014). The cAMP chemotactic signaling pathway of *D. discoideum* cells has been well-characterized and provides a general concept for how gradient information is processed to initiate directional motility (Swaney et al., 2010; Artemenko et al., 2014; Tang et al., 2014; Nichols et al., 2015). cAMP binds to cAMP receptor 1 (cAR1), a member of the GPCR family, to activate its cognate heterotrimeric G protein (G protein), making cAR1 a guanine exchange factor (GEF). The activation is transmitted to several redundant downstream pathways including phosphatidylinositol-3,4,5-trisphophate (PIP3), TorC2-PDK-PKB, 3’,5’-cyclic guanosine monophosphate (cGMP), and phospholipase A2 (Pla2) signaling. PIP3 production and TorC2-PDK-PKB activity are mediated by the localized activation of RasG and RasC, respectively, at the front of the cells, resulting in pseudopod formation. The cGMP pathway regulates myosin II to generate contractile force at the back (Bosgraaf et al., 2005; Veltman and Van Haastert, 2007), while one recent report suggested an additional negative role of the cGMP pathway in the depolymerization of f-actin at the front (Tanabe et al., 2018). Importantly, each signaling pathway displays an intrinsic excitable property with the all-or-none response to a suprathreshold stimulus (Arai et al., 2010; Nishikawa et al., 2014; Fukushima et al., 2019; Li et al., 2020). In addition to excitability, PIP3 is produced in a bistable manner (Matsuoka and Ueda, 2018). Thus, shallow gradient signals can be amplified for a cell to output and sustain the constant activity of each signaling molecule at the front.

However, recent research on *D. discoideum* cells has indicated that additional G protein dynamics in response to cAR1 activation occur in chemotaxis. Here we describe these new dynamics after reviewing classical chemotaxis G protein signaling in *D. discoideum* cells as a model for eukaryotes.

### Overview of cAMP Chemotactic GPCR Signaling

**FIGURE 1:**
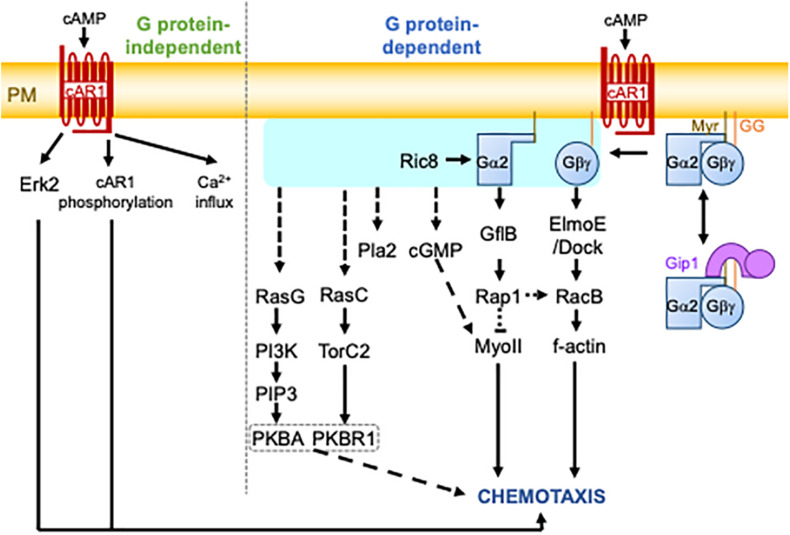
The cAMP chemotactic signaling pathway in *D. discoideum* cells. Upon cAMP binding, the cAR1 receptor triggers heterotrimeric G protein-independent and -dependent pathways. The G protein-independent pathway involves Erk2 activation, cAR1 phosphorylation, and calcium (Ca^2+^) influx. The G protein-dependent pathway involves the activation or dissociation of G proteins catalyzed by the GEF activity of cAR1. The activation of G proteins is shown by the light blue box. This is followed by the activation of several signaling molecules, finally leading to chemotaxis. It is still unknown how G protein activity is transmitted to some signaling molecules, including RasG, RasC, Pla2, and cGMP. G proteins are comprised of Gα2 and Gγ subunits, which are myristoylated (Myr) and geranylgeranylated (GG), respectively, for their plasma membrane localization. They undergo complex formation with Gip1 in the cytoplasm, which allows for the spatial regulation of G proteins. The dashed lines represent several or unknown steps.

cAMP-induced chemotaxis is one of the most common experimental settings for studying chemotaxis and is a framework of the eukaryotic chemotaxis mechanism (Swaney et al., 2010; Figure 1; the major factors are summarized in Table 1). When environmental nutrients are depleted, solitary *D. discoideum* cells start the developmental process to make a multicellular structure. In early development, some cells produce cAMP, which is relayed to other cells through a process known as cAMP signal relay to make periodic cAMP propagating waves. The waves can guide about a hundred thousand cells into an aggregate via chemotaxis. During this aggregation, cells form migration streams in which they align their morphological directionality and attach to each other side by side. cAMP binds to a series of receptors, cAR1 – cAR4, with different affinities (Kim et al., 1996). cAR1 is the major receptor for cAMP chemotaxis in early development. It binds to cAMP with two different affinities: a high affinity of 3.5∼30 nM and a low affinity of 200∼500 nM (Van Haastert, 1984). It is known that cAR1 transduces signals in G protein-dependent and -independent manners. GPCR-independent events include extracellular calcium influx and Extracellular signal-regulated kinase 2 (Erk2) activation (Milne and Devreotes, 1993; Milne et al., 1995; Brzostowski and Kimmel, 2006). Erk2 has been associated with cAMP chemotaxis because of its role in folate chemotaxis (Nichols et al., 2019). The role of G protein, which is comprised of the subunits Gα2 and Gβγ, in cAMP chemotaxis is less controversial. There are twelve Gα subunits in *D. discoideum* cells, whereas Gβγ is encoded by a single gene. Gα2 is myristoylated at the N terminus, and Gγ is geranylgeranylated at the C terminus (Miyagawa et al., 2018). These lipid modifications ensure the localization of G proteins to the plasma membrane. cAR1 catalyzes a guanine exchange reaction of the α subunit of the Gα2-Gβγ complex. The GTP bound form of Gα2 forces the dissociation of Gβγ. The *Dictyostelium* homolog of Resistance to inhibitors of cholinesterase 8 (Ric8) was isolated as a binding protein to Gα and shown to facilitate a GEF-reaction of Gα independently of cAR1 (Kataria et al., 2013). Ric8-mediated G protein activation is especially required for chemotaxis at lower cAMP concentrations. The dissociated subunits transmit information of an ambient chemical gradient on the plasma membrane. Another Gα2 binding protein, GEF like protein B (GflB), serves as an effector of Gα2 to regulate the cytoskeleton through Ras and Rap1 (Liu et al., 2016). GflB is also a RacE-binding protein (Senoo et al., 2016). Furthermore, GflB coordinates the activities of Ras and Rho for proper gradient sensing. ElmoE binds to Gβγ and serves as a GEF for RacB with Dock-like proteins to form f-actin (Yan et al., 2012).

**TABLE 1:** GPCR signaling and its related factors in the cAMP chemotactic signaling pathway.

**Gene (protein)**	**Gene ID (DDB number)**	**Molecular feature**	**Mammalian homolog**
cARA (cAR1)	DDB_G0273397	GPCR	No cAR1 homologs but various GPCR serve for chemotaxis, e.g., formyl-peptide receptor and CXCL8 receptor
gpaB (Gα2)	DDB_G0276267	Heterotrimeric G protein α subunit	Gαi
gpbA (Gβ)	DDB_G0277143	Heterotrimeric G protein βγ subunit	Gβ
gpgA (Gγ)	DDB_G0274125	Heterotrimeric G protein βγ subunit	Gγ
adcB (AdcB)	DDB_G0274395	Arrestin	β-arrestin
adcC (AdcC)	DDB_G0271022	Arrestin	β-arrestin
ric8 (Ric8)	DDB_G0292036	Non-receptor GEF	Ric8
gipA (Gip1)	DDB_G0271086	G protein shuttling factor	TNFAIP8 family proteins
gflB (GflB)	DDB_G0286773	Gα2 binding factor, RapGEF, RasGEF, RhoGAP	
elmoE (ElmoE)	DDB_G0279657	Gβγ binding factor, RacGEF as the Elmo/Dock complex	ELMO1

cAR1 undergoes multiple phosphorylations in the C terminus cytoplasmic region upon cAMP stimulation, although the corresponding kinase is still unknown (Hereld et al., 1994). The phosphorylation shifts the low cAMP binding affinity from ∼300 to 800 nM, suggesting that the chemotactic dynamic range moves toward lower concentrations (Caterina et al., 1995). This hypothesis was tested using a non-phosphorylatable mutant of cAR1. The mutant still showed chemotaxis even in higher concentration ranges, though the efficiency was reduced (Kim et al., 1997). Thus, phosphorylation could be required for attenuating the cAMP signal pathway, such as adenylyl cyclase and Erk2 activities, and for secondary actin polymerization (Brzostowski et al., 2013). In mammalian cells, phosphorylated cytoplasmic residues of GPCR recruit Arrestin, which competes with G protein dependent signaling, leading to internalization. *D. discoideum* has six genes, *adcA-F*, which encode arrestin domain-containing protein. AdcB and AdcC have redundant functions in the early development (Cao et al., 2014). AdcC associates with cAR1, the phosphorylation of which increases the efficiency of the association. AdcB and AdcC are required for cAR1 internalization under high cAMP concentrations and longer times than in the effective ranges for chemotaxis at early development. Consistently, cells lacking AdcB and AdcC show normal chemotaxis. These results suggest that receptor internalization does not extend the chemotactic dynamic range.

*D. discoideum* cells can sense spatial information in cAMP gradients. Cells treated with latrunculin become round because of f-actin depletion. When such immotile cells are stimulated by cAMP gradients, downstream signaling, such as PIP3 production and Ras activation, are transiently activated along the entire membrane (Parent et al., 1998; Xu et al., 2005; Kamimura et al., 2016). Subsequently, the activation is biased at the higher concentration side of the gradient. In the whole process, G proteins are persistently activated at different levels at the front and back of the cells (Xu et al., 2005). Therefore, G protein activity reflects the local concentration along the gradient.

### cAR1 Activates Heterotrimeric G Protein Gα2-Gβγ

As described above, cAR1 activates heterotrimeric G protein through its GEF activity, in which Gα2 changes GDP to GTP and simultaneously dissociates from Gβγ. The activation has been visualized *in vivo* by fluorescence resonance energy transfer (FRET) experiments in which Gα2 and Gβ are fused to cyan and yellow fluorescent proteins, respectively (Janetopoulos et al., 2001). Before cAMP stimulation, the heterotrimeric form of Gα2-Gβγ showed FRET signals, but after the stimulation the FRET was lost, reflecting the dissociation into Gα2 and Gβγ. We recapitulated these results using a combination of Gα2-Cerulean and Gβ-Venus, the activation of which had an EC_50_ = 2.3 nM, finding G protein activation is saturated at a lower concentration than at which *D. discoideum* cells show chemotaxis (Miyanaga et al., 2018). The full activation of G proteins at lower cAMP concentrations was explained by a model in which ligand-bound receptors continuously activate G proteins (Xu et al., 2010).

### Gip1-Mediated G Protein Translocation

G protein interacting protein 1 (Gip1) was identified as a binding protein of Gβγ by a biochemical tandem affinity purification experiment in *D. discoideum* cells (Kamimura et al., 2016). It has an N-terminal Pleckstrin-homology (PH) domain and C terminus that is weakly similar to mammalian tumor necrosis factor α induced protein 8 (TNFIP8). A deletion analysis showed that its C terminus is sufficient for binding to G proteins. However, its full-length, which includes the PH domain, is required for its physiological function based on rescue experiments of the early developmental phenotype. *gip1Δ* cells show small aggregates at early development upon starvation, whereas wild-type cells make streams by chemotaxis. Interestingly, *gip1Δ* cells lose chemotactic ability, especially at higher cAMP concentrations. G proteins localize in the cytosol as well as the plasma membrane in wild-type cells. In contrast, *gip1Δ* cells lose cytosolic G proteins but not membrane ones. These results indicate that G proteins shuttle between the plasma membrane and the cytosol, consistent with previous observations (Elzie et al., 2009). Cytosolic G proteins bind to Gip1. Moreover, cytosolic G proteins translocate to the plasma membrane upon cAMP stimulation in a Gip1-dependent manner, with an EC_50_ from the cytosol of 10 nM. The translocation still occurs without Ras activation, PIP3 production, or f-actin, suggesting unknown upstream signaling. The dynamic spatial regulation ensures the redistribution of G proteins at the plasma membrane along ambient cAMP gradients. This redistribution is required for gradient sensing at higher concentration ranges.

The crystal structure of the C terminus of Gip1 can help clarify the mechanism underlying its complex formation with G proteins (Miyagawa et al., 2018). The G protein binding region of Gip1 is composed of six α-helices which fold into a cylinder-like structure with a central hydrophobic cavity. The cavity is 22 Å in depth and 10 Å in diameter and includes glycerophospholipids derived from bacteria for Gip1 overproduction. Experiments on the binding site of G proteins have also been performed. Biochemical and genetic analyses revealed that the geranylgeranyl modification on Gγ is essential for the complex formation. This result suggests that the hydrophobic cavity of Gip1 accommodates the lipid modification of Gγ in the cytosol. Consistently, when steric hindrance is introduced into the cavity by replacing the amino acid residues making the cavity with tryptophan, the binding to G proteins deteriorates. Collectively, a model for G protein shuttling has been proposed. In the resting state, G proteins are not permanently anchored on the plasma membrane. Therefore, some detach from the membrane, resulting in cytosolic sequestration by interacting with Gip1. This interaction involves the hydrophobic cavity of Gip1 and the lipid modification of G proteins, stabilizing the complex in the hydrophilic environment of the cytosol. Chemoattractant signaling causes G proteins to dissociate from Gip1 by a plausible conformational change of the hydrophobic cavity. The N-terminal PH domain of Gip1 may regulate the configuration of the cavity upon cAMP stimulation.

### Stable Complex Formation Between Activated Gα2 and cAR1 Receptor

We established a single molecular imaging technique to analyze the binding of fluorescently labeled cAMP to *D. discoideum* cells in chemotaxis (Ueda et al., 2001). This technique has since been applied to cAR1, Gα2, and Gγ to show their molecular dynamics upon cAMP stimulation (Miyanaga et al., 2018). These molecules were fluorescently labeled by tetramethylrhodamine (TMR) via HaloTag. TMR is a small organic fluorescent dye and shows a stronger signal and longer fluorescence longevity than fluorescent proteins, thus providing better data (Miyanaga et al., 2009). All molecules showed free diffusion despite their distinct diffusion coefficients. While cAR1-TMR has a single diffusion coefficient of 0.017 μm^2^/s in the absence of cAMP, Gα2-TMR and Gγ-TMR have two diffusion coefficients of 0.016 and 0.20 μm^2^/s and of 0.029 and 0.21 μm^2^/s, respectively. When cells are stimulated with saturating cAMP concentration (10 μM), neither cAR1-TMR nor Gγ-TMR changed their motility, but the slower fraction of Gα2-TMR increased from 20 to 53%. A dose-dependent study revealed that the shift to the slower fraction had an EC_50_ of 270 nM. There are two points we want to highlight from these studies. First, the diffusion coefficients of cAR1-TMR and the slower fraction of Gα2-TMR are similar; and second, the EC_50_ of 270 nM matches the lower affinity of cAR1 receptors for cAMP. These findings indicate that activated Gα2 interacts with low-affinity cAR1 to make a stable complex and slows down in the plasma membrane.

To test this hypothesis, Gα2-TMR mobility was observed upon the external modulation of cAR1. Benomyl, an inhibitor of tubulin polymerization, is known to reduce cAR1 motility (de Keijzer et al., 2011). When cells were treated with benomyl, Gα2-TMR showed slowed cAMP-stimulated motility based on the diffusion coefficient changing from 0.015 to 0.005 μm^2^/s, which was concomitant with the decrease in the cAR1-TMR diffusion coefficient. In these experiments, cAR1 is tethered to a glass surface through biotin and avidin. Thus, cAMP stimulation slows the slower mobile fraction of Gα2-TMR such that the diffusion coefficient is almost the same as that of tethered cAR1, supporting the hypothesis. Additionally, experiments using a constitutively active form of Gα2 with a Q208L mutation revealed that the cAMP-stimulated slow mobile fraction includes the activated, or GTP-bound, form of Gα2, since this mutant also showed the shift to the slow mobile fraction.

The stable complex formation provides a mechanism for gradient sensing at higher concentration ranges (Miyanaga et al., 2018). Furthermore, single molecular analysis techniques can measure membrane binding lifetimes. The lifetime of Gα2-TMR prolongs after cAMP stimulation, but the lifetime of Gγ-TMR does not. When a cell is exposed to a steep gradient, the side facing the higher cAMP concentration has a longer lifetime with slower mobility than the side facing the lower concentration. This feature relates to the stable complex formation between low-affinity cAR1 receptors and activated Gα2, which has an extended lifetime. Thus, cAMP concentrations are affected by the generation of intracellular G protein gradients along cAMP gradients at higher concentration ranges.

### Three Different G Protein Dynamics for Broad Range Chemotaxis

**FIGURE 2:**
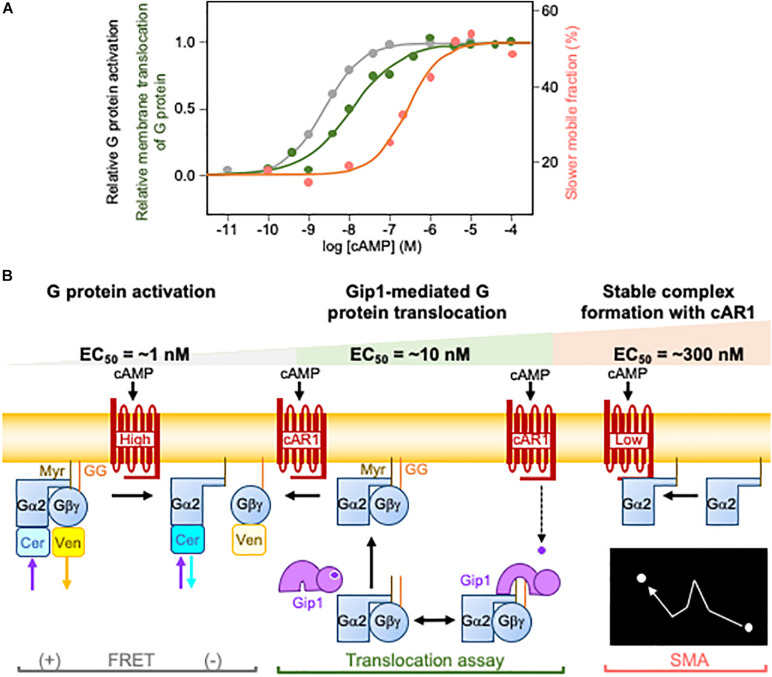
Three different G protein dynamics for cAMP wide-range chemotaxis. **(A)** The three different G protein dynamics include G protein activation, Gip1-mediated G protein translocation, and a slower mobile fraction caused by the stable complex formation with cAR1. Each has a distinct EC_50_ shown in black, green, and red, respectively. **(B)** Schematic illustration of the three different G protein dynamics. G protein activation, Gip1-mediated G protein translocation, and the slower mobile fraction caused by the stable complex formation. Experimentally, they are assessed by fluorescence resonance energy transfer (FRET) between Gα2 with Cerulean (Cer) and Gβ with Venus (Ven), the translocation of G proteins upon cAMP stimulation, and single molecular imaging analysis (SMA), respectively.

The quantitative characterization of chemotaxis has shown that *D. discoideum* cells detect a chemical gradient over broad ranges of average concentrations (Mato et al., 1975; Fisher et al., 1989; Song et al., 2006; Ohtsuka et al., 2021). Cells can move up cAMP gradients as small as sub-nM to as large as μM. How cAMP gradient information is converted into intracellular gradients for G protein signals is unknown, however. cAR1 receptors activate G proteins as GEF in cAMP gradients but saturate at a lower concentration than at which cells show chemotaxis (Janetopoulos et al., 2001; Miyanaga et al., 2018). Therefore, *D. discoideum* cells must rely on another mechanism to produce an intracellular gradient of G protein activity. High-affinity cAR1 can likely activate G proteins, as it has an EC_50_ that is comparable to high-affinity cAR1 for cAMP. However, the fraction of high-affinity cAR1 is estimated to be about 10% (Milne et al., 1997), meaning we still lack understanding for the other 90% of cAR1, which is low-affinity. Three distinct G-protein dynamics offer an explanation for wide-range chemotaxis (Figure 2). In the first, cAR1-catalyzed G protein activation occurs at an EC_50_ of 2.3 nM (Miyanaga et al., 2018). In this concentration range, high-affinity cAR1 produces biased G protein activity along the cAMP gradient. In the second, in increasing cAMP concentrations, Gip1-mediated G protein translocates with an EC_50_ of about 10 nM to recruit G proteins from the cytosol to the membrane (Kamimura et al., 2016; Miyanaga et al., 2018). The spatial regulation of G proteins facilitates their distribution along the cAMP gradient. If this distribution does not occur, which is the case in *gip1Δ* cells, chemotactic cells cannot discern the proper direction because the G protein activity is already saturated. In the third, further increases in cAMP concentration trigger the stable complex formation of activated Gα2 and cAR1 with an EC_50_ of 270 nM, which is similar to the Kd for low-affinity receptors (Van Haastert, 1984; Miyanaga et al., 2018). Therefore, at higher concentration ranges, low-affinity receptors provide cAMP gradient information to G proteins through physical interactions. Collectively, these three G protein dynamics mechanisms cover the whole chemotactic dynamic range, from the sub-nanomolar to several micromolar, of cAMP.

## Conclusion and Future Perspectives

Broad range chemotaxis is important for the physiology of *Dictyostelium* cells and human cells; for example, starved *Dictyostelium* cells and mammalian neutrophils are efficiently recruited to the center of aggregates and to tissue damage or infection sites from the blood circulation, respectively. Although it was shown more than 30 years ago that chemotactic cells show directional movement over broad ranges, the mechanism has only been revealed in the past several years using *D. discoideum* cells as a model organism. Key findings include distinct G protein dynamics in response to distinct chemoattractant concentrations. How GPCR generally activates G protein by its GEF activity is well characterized (Weis and Kobilka, 2018). However, the intracellular spatial regulation of G proteins has received less attention. The identification of Gip1 in *D. discoideum* cells provides the molecular basis of the spatial regulation, but many questions remain (Kamimura et al., 2016; Miyagawa et al., 2018). How does Gip1 bind to the trimeric form of G proteins? Does the spontaneous dissociation of G proteins require any factors? What is the signaling molecule target for the PH domain of Gip1 to break the complex formation with G proteins? And, how are G proteins stabilized on the plasma membrane upon cAMP stimulation? Furthermore, it is unknown if the mechanisms for G protein dynamics is conserved in mammalian cells. Some evidence supports conservation. For example, G proteins shuttle between the plasma membrane and organelles in mammalian cells (Saini et al., 2009). Also, the binding region of Gip1 has homology with the mammalian TNFAIP8 family. TNFAIP8 proteins have a hydrophobic cavity like Gip1 and have been reported to participate in immunity and various diseases (Goldsmith et al., 2017; Niture et al., 2018; Zhang et al., 2018). Collectively, TNFAIP8 may regulate the spatial regulation of G proteins for the broad range chemotaxis of neutrophils. Another G protein dynamics found in *D. discoideum* cells by single molecular analysis is the stable complex formation mechanism (Miyanaga et al., 2018). Activated Gα2 but not the trimeric form of G proteins is responsible for this formation. The result indicates that the stable complex is formed by yet unidentified binding sites of Gα2 and cAR1. This mechanism has a unique feature in that the low-affinity fraction of cAR1 receptors provides the binding site for activated G proteins to make intracellular gradients. Given that GPCR signaling is conserved in eukaryotes, the same mechanism may serve in mammalian cells.

Chemotaxis is comprised of many factors operating in a complex network for stable directional motility in fluctuating environments. To understand how this network conducts gradient sensing, several mathematical models have been proposed in *D. discoideum* cells, including Local Excitation and Global Inhibition (LEGI) (Parent and Devreotes, 1999; Iglesias and Devreotes, 2008). The three types of G protein dynamics described here have still not been incorporated into such models, however. For that, more quantitative parameters of G protein dynamics are needed. In addition to such G protein dynamics, cAR1 phosphorylation influences the chemotactic dynamic range (Kim et al., 1997), and the RasGAP C2GAP1 regulates the chemotactic range (Xu et al., 2017). These reactions should be included in models for the complete understanding of broad range chemotaxis.

Finally, studies on eukaryotic chemotaxis using *D. discoideum* cells as a model system have provided insights into the mechanism of not only broad dynamic range chemotaxis but also GPCR signaling. Especially, the spatial and temporal regulation of G proteins has been found in *D. discoideum* cells as a novel mechanism of GPCR signaling. However, more structural and biochemical analyses are required for complete understanding of the mechanism and its physiological significance. Such analyses could give way to new therapeutic targets for human diseases derived from compromised chemotaxis and more broadly advance GPCR-related biology.

## Author Contributions

YK and MU conceptualized the contents, revised the manuscript, and approved the submitted version. YK wrote the initial draft of the manuscript and prepared the figures. Both authors contributed to the article and approved the submitted version.

## Conflict of Interest

The authors declare that the research was conducted in the absence of any commercial or financial relationships that could be construed as a potential conflict of interest.

## Publisher’s Note

All claims expressed in this article are solely those of the authors and do not necessarily represent those of their affiliated organizations, or those of the publisher, the editors and the reviewers. Any product that may be evaluated in this article, or claim that may be made by its manufacturer, is not guaranteed or endorsed by the publisher.
